# What Does a Systems Approach to Quality Improvement Look Like in Practice?

**DOI:** 10.3390/ijerph19020747

**Published:** 2022-01-10

**Authors:** Sharon J. Williams, Stephanie Best

**Affiliations:** 1 School of Health and Social Care, Swansea University, Swansea SA2 8PP, UK; 2 Australian Institute of Health Innovation, Macquarie University, Sydney, NSW 2109, Australia; Stephanie.best@mq.edu.au

**Keywords:** health systems, quality improvement, QUASER, process improvement, systems thinking, NHS

## Abstract

Universally improving healthcare systems is difficult to achieve in practice with organisations implementing a range of quality improvement (QI) approaches, in varying and changing contexts, and efforts ranging from project-based improvements to whole system change. This study aimed to identify how organisations overcome the challenges to improving the quality of the services they deliver. Drawing on the eight challenges from the ‘Quality and Safety in Europe by Research (QUASER) hospital guide, we assessed eight cases reported by the UK-based regulator Care Quality Commission as improving their performance. A thematic analysis of these secondary data established that all eight challenges had been addressed or considered in varying degrees. Education and physical and technological challenges seemed less prominent than developments made to address other challenges such as developing leadership, structure, and culture to support improving quality. This paper relies on the analysis of secondary case data and one framework to assess improvement efforts. Further research is required to consider other models and frameworks and to collate longitudinal data to capture the dynamics and increasing the maturity of improving healthcare systems in practice.

## 1. Introduction

Implementing a whole of system approach to quality improvement (QI) is a practice many organisations are striving to achieve but at the same time seems to be nebulous as to how it might be achieved or whether it has been achieved. This systems approach seems to be the ‘pot of gold’ at the end of a rainbow. Several reviews of improvement approaches such as that by Lean have analysed and categorised improvement efforts as mainly ranging from point of care-based projects to a whole hospitals/system approach [1] with the former being more frequently cited. There is a wealth of information about the tools and techniques that need to be employed to make improvements [2,3]. However, we need to move beyond the implementation of tools and as Braithwaite [4] has argued, we need to understand why system-wide progress has remained so elusive and difficult to achieve and to identify which improvement initiatives have made positive contributions to improving healthcare systems. Similarly, Kaplan et al. [5] noted the widespread efforts of implementing quality improvement in healthcare, but also mention the significant variability of success.

The aim of this paper was to try and shed some light on what challenges organisations in healthcare face and how these are overcome when trying to improve the quality of their service and move to a systems approach for improvement. Using published case studies of a small sample of improving National Health Service (NHS) trusts in England, we analysed these secondary data to understand:

- What challenges have been addressed to improve their performance?
- Are some of these challenges more prominent that others?
- Is there any evidence that improvements are at a systems level?

## 2. Literature Review

### 2.1. What Is Systems Thinking?

Before we consider this question in relation to improving healthcare services, it is useful to consider what we mean by systems thinking. There is considerable coverage and in the literature across various disciplines that contribute to our understanding but at the same time there is a continual debate concerning where a system may start and finish, what level it might operate. Cusins [6] provides some simple definitions that we believe are helpful to understand this concept. First is to define the system from its environment by identifying an arbitrary boundary (e.g., acute care, hospital wide). Second, which inputs from its environment cross the boundary into the system (e.g., patients, information, supplies). Third, within the system, be clear regarding how the inputs interact in a transformation process (e.g., surgery). Fourth, how do the transformed inputs leave the system as outputs (e.g., patients, information). This can also be seen as an input–output model operating within an arbitrary boundary. Having agreement about the scope of the system and its defined inputs, transformation process and outputs will help us understand how system change might be achieved. Often, our improvement efforts now need to span an integrated care system where the boundary is drawn across several services such as acute and primary care. 

### 2.2. A Systems Approach to Healthcare Improvement

A systems approach to healthcare has been gathering increased attention since the first half of the 20th century [7]. There have been several high-profile reports published in the USA [8,9] and the UK [10,11] that significantly challenge the status quo and recognise the need for a better approach to improving the quality of the healthcare delivery systems, along with the realisation that healthcare organisations are complex, adaptive systems which has implications for change [9]. In the UK, this led to the first review of design and systems practice within healthcare [12]. Since then, both in the USA and the UK, there have been many reports that have highlighted the importance of using a systems approach to improving the design and delivery of healthcare, e.g., [13,14,15,16].

Prior to and since the publication of these key reports advocating for change in the delivery of care, quality and safety issues persist [2]. In an attempt to mitigate such issues and raise the importance of quality improvement with hospitals boards, there have been several studies informing us what needs to be in place to support a whole hospital or systems approach to improve healthcare. For example, one of the early studies by Shortell et al. [17] assessed the evidence relating to the impact of continuous quality improvement on clinical practice, which could be used to help accelerate progress. Three conditions were identified from this study in which QI applications were likely to be more effective which were as follows:

- Application of QI to focus on areas of priority with carefully designed interventions;
- The organisation is prepared and ready for change, including capable leadership, good relations with staff, and supportive information systems;
- The external environment is conducive relative to beneficial regulatory, payment policy and competitive factors.

A more recent survey-based study of over 1000 US hospitals found that only 12% of hospitals reported being at a mature hospital-wide stage of implementation, which was reported to rely on leadership commitment, the use of a daily management system and training in quality improvement [18]. A study of 15 healthcare organisations in England reported that those with high maturity in quality improvement (QI) had boards that prioritised QI, balanced short-term priorities with long-term investment in QI, used data for improvement not just quality assurance, ensured patients and staff were engaged in their QI work and promoted a culture of continuous improvement were more likely to be successful with their QI efforts [19]. 

To further understand and conceptualise our understanding of what constitutes a systems approach in the practice of improving healthcare services, we used a small sample of NHS hospitals in England, UK, which have notably improved their performance and have mostly been rated by the regulator, the Care Quality Commission (CQC), as good or outstanding. Several of these cases were reported as part of their improvement journey to be looking at their involvement in the local wider health and care systems [20]. To guide our assessment of these case hospitals, we draw on the internationally renowned ‘Quality and Safety in Europe by Research’ (QUASER) guide to understand what organisations are doing in practice and if they are addressing the key challenges of improving their performance and moving to a systems approach. 

The QUASER study builds on the “Organising for Quality Study published in 2008 [21] by identifying six common challenges which high-performing hospitals have recognised and successfully addressed, which were later extended to eight [22]. These eight challenges are summarised in Table 1. The starting point for this original study was recognising that whilst technical factors, such as information systems, play an important role in accounting for the quality ‘gap’, organisational and cultural factors are also crucial in understanding how quality and safety improvements are achieved [3,22]. There has been a growing interest in the role of hospital leaders and boards in the quality of care [23,24,25]. Core responsibilities of the management or executive boards of hospitals include defining and setting strategy, advising on management, evaluating performance and providing oversight and control [26]. The aim of this study was to investigate how hospitals implement, spread and sustain quality improvement to establish the difficulties in doing so and how organisations endeavoured to overcome these challenges [3]. The QUASER guide was developed to facilitate systematic and detailed discussions amongst senior hospital leaders about organisation (system)-wide strategies for improving QI. 

We recognise that there are many other conceptual models and frameworks that were published to help organisations to prepare and understand the success of their improvement efforts. For example, the popular Model for Understanding Success in Quality (MUSIQ) [5] established 25 key contextual factors that are likely to impact QI success, which can be used by researchers and implementers to study how context influences QI success. Reed et al. [28] further developed this model by identifying three additional factors. The original MUSIQ is also reported to be well aligned with other popular related models and frameworks such as the Promoting Action on Research Implementation in Health Services (PARiHS) framework [29,30], Consolidated Framework for Implementation Research (CFIR) [31] and the Agency for Healthcare Research and Quality (AHRQ) [32,33,34]. Clearly, the application of all these models is beyond the scope of this paper. QUASER seems to encompass many of the factors highlighted within these alternative models/frameworks. In addition, QUASER is based on the dialogue of the senior management team and managers. It is these groups that have informed the published cases that we examined in this study.

## 3. Study Context

CQC, the regulator of health and adult social care service providers in England, checks through inspection and ongoing monitoring, that government standards are being met by providers such as hospitals, care homes and general practitioners (GPs). When services are being inspected, five fundamental questions are asked:

1. Is it safe? Are patients protected from abuse and avoidable harm?
2. Is it effective? Does the care, treatment and support provided achieve good results and help to maintain quality of life and is the care based on the best available evidence?
3. Is it caring? Are patients involved in their care, are they treated with compassion, kindness, dignity and respect?
4. Is it responsive? Are services organised so that these meet the needs of the patients?
5. It is well led? Does the leadership of the organisation make sure high-quality care is provided and is it patient centred? Does it encourage learning and innovation and promote and open and fair culture? [35].

After each inspection, a report is published on the CQC website. The report provides the answers to these five key questions and describes areas of good practice and any concerns. The ratings awarded range from inadequate, requiring improvement, good and outstanding (see CQC for more details on aggregate ratings). Up until recently, NHS trusts could be placed into special measures if problems relating to quality and safety and/or leadership. More recently, this has been replaced by the Recovery Support Programme. NHS England and NHS Improvement used the CQC ratings to help decide whether NHS trusts require extra support to address issues of finance or quality of care. In this study, we selected all of the case organisations in 2017 that were highlighted by CQC as significantly improving their performance, with the intention to understand whether the success of these organisations was due to pursuing a systems approach to improving their services. At the time that these cases were published, ‘special measures’ were still in place. Special measures were first introduced because of the Keogh [36] report which investigated 14 NHS trusts with high mortality rates. Although the report found elements of excellent practice in all trusts, there was significant scope for improvement. Eleven of the fourteen trusts were placed under ‘special measures’. In 2018, there were 20 trusts in England in special measures, seven for financial reasons, eight for quality reasons, and five for both quality and financial reasons [37]. At that time, there were approximately 223 trusts which include acute, mental health, specialist or community trusts ([38]. It is important to note that the number of NHS trusts does not represent the number of hospitals as many trusts run more than one hospital. More recent figures show there are four healthcare systems and 16 trusts (12 of which were under special measures) that were placed into the new “recovery support programme” [39].

## 4. Materials and Methods 

### 4.1. Selection of Case Studies

The eight cases included in this analysis were purposively selected from those published on the CQC’s website as significantly improving their performance and ratings by the regulator. There were five foundation trusts, four university trusts, one teaching foundation trust, one trust and one labelled as an NHS hospital which is part of a foundation trust. Table 2 reports on the key characteristics for each organisation including population served, staff numbers and the number of beds.

Five trusts improved by two ratings, and three trusts improved by one rating [35]. The cases were compiled by the CQC after conducting a series of interviews within each organisation; interviewees included the chief executives, medical and nursing directors, patient representatives and a range of external stakeholders.

For our analysis of these published cases, we were interested to see whether there was any evidence that the case organisations, described as ‘well led’ by the CQC, had improved their performance by addressing the eight challenges identified in the QUASER study. We employed this framework to guide and format our data collection.

### 4.2. Data Analysis

First, the eight case studies were framed with common themes of improvement: reaction to initial inspection report/rating; leadership; cultural change; vision and values, governance, improving safety, public and patient involvement; looking outwards; and CQC engagement.

Using NVivo 12 textual analysis was conducted on the case narrative using the thematic analysis and comparing the data with the QUASER criteria (see Table 1). The qualitative accounts published by CQC were precisely selected—indeed, purposively—to illuminate potentially important differences and similarities [40]. This was an iterative process where the data for each case were analysed and comparisons then drawn across all eight cases in relation to whether QUASER criteria were evident from the published CQC report [20]. QUASER is a research-based framework intended as a reflective tool for analysis to help identify the strengths and weaknesses of improvement efforts and to recognise where further improvements are needed. Here, we are using the guide to retrospectively consider whether the improvements made by the case organisations were achieved by addressing some or all of the eight QUASER challenges.

## 5. Results

Here, we present a summary of the case organisations selected by the CQC [20] before reporting how each of the QUASER challenges were represented in practice. Table 3 illustrates the status for each of the case organisations from being rated as inadequate or requires improvement, and for most, achieving good or outstanding.

Evidence of each QUASER QI challenge was identified in most case studies (see Table 4). The educational challenge was the most commonly missing QUASER code (n = 4) followed by physical and technical (n = 2) and finally, structural (n = 1). The challenges are mainly associated with system inputs (I). We considered leadership as being an input and a transformation process as it will also shape the performance of the organisation. Similarly, we classified emotional challenges as a transformation process. None of the challenges were directly linked to outputs of the system but indirectly all of the challenges are likely to impact on patients, staff and their relatives. 

### The Eight Challenges of Quality Improvement

Each challenge identified in the QUASER study is individually reported with examples of how it was met by the case organisations. However, as noted by Anderson et al. [22], many of the areas overlap; for example, there is a close interdependence between leadership and culture.

1. **Leadership:** The CQC described the case organisations as well led [20]. Therefore, it was envisaged that this challenge would be met by all the organisations. The role of leadership was indeed seen across the organisations and influences many of the eight QUASER quality improvement challenges and the transformation process. Providing a common purpose was reported to be helpful:

“The trust used the CQC inspection as a lever for clinical improvement… “we needed to get people into a room to talk together, to develop a solution.”

(Case 8)

Being seen, physically being seen and making leaders accessible to staff was described in one case as essential:

“A priority … across the trust is making sure that she [chief nurse] is visible and accessible, and communicating well with staff. She has weekly meetings with senior ward managers, matrons and divisional nurses to keep her “in touch with the shop floor all of the time”.

(Case 6)

There were approaches to gathering intelligence about how the organisation is doing:

The communications team put together a full range of activities to keep staff, patients and stakeholders informed from day one. To find out the main issues for staff, {comms team] used surveys and staff focus groups. “We talked to all staff groups to get a better understanding of the challenges,” …. “Their feedback enabled us to highlight the major issues around the trust and specific areas where we could support improvement.”

(Case 3)

Importantly, staff within the organisation needed to know what they were working towards:

The Chief of Service leaders, along with the executive team, started to make it clear what ‘good’ looked like through sheer commitment and determination. This element showed staff that senior clinical and managerial leaders were committed to staff and patients. Staff started to recognise that improvement was needed, and that they could make a real difference.

(Case 4)

2. **Political**: The importance of widely engaging with stakeholders was reported as being important along with sharing key quality improvement messages:

“The trust kept staff and patients at the heart of discussions when shaping governance and processes. They kept the idea in mind: If this was your family member or friend, would the care be good enough? The trust also surveyed staff and patients to understand how people felt things were progressing.”

(Case 4)

Specific initiatives included, ‘In Your Shoes’ listening events (Case 8) and ‘Enter and View’ visits to the hospitals (Case 7) with engagement reported as an ongoing activity rather than a one-off event:

XX is one of the parents involved in helping to shape the design of the trust’s new maternity unit. She says that local people were fully involved in the development by meeting the architect and contractors, looking at the options and making suggestions. The trust acted on the parents’ suggestion of using the bereavement room as a place to stay for families with a terminally ill child or a child receiving special care.

(Case 1)

3. **Cultural**: Building shared values was critical to quality improvement success. Staff were encouraged to share how they felt about the organisation and their roles:

The new leadership team encouraged people to say how they felt about the trust. Staff were encouraged to talk about their roles, what they felt was positive and what stopped them delivering great care. An online tool called Wayfinder was used to get staff involved and engaged in developing the trust’s values, called the ‘XXXX (hospital name) Way’.

(Case 7)

Leadership was a key component of creating a positive quality improvement culture, particularly one being visible to staff:

“Before, some staff didn’t know who the chief nurse was–they knew the name, but had hardly spoken to them. Now you see the management team on the floor, actually walking through your door and saying, ‘well done team–thanks for all your hard work.’ Two or three little words make a massive difference for staff.”

(Case 8)

Additionally, staff were empowered to take control of their area and their quality improvement, which was supported by executive sponsors:

“We worked with them to get them to control their own areas. We provided training for managers and non-executives mentored people and divisions.”

(Case 1)

4. **Educational**: Organisations reported the provision of educational activities to upskill their staff in quality improvement with direct application to their day-to-day practice:

Throughout 2014, the trust provided learning sessions and a summit to engage staff in a new quality improvement methodology aimed at reducing pressure ulcers.

(Case 2)

Again, the role of leadership was seen as central, here in relation to the ongoing impact of educational activities:

“…the new clinical lead for the tissue viability team set up training and education for ward staff, which “empowered staff to engage more with the service”. The clinical lead worked on recognising the tissue viability needs of patients on admission and improving incident forms, documentation and reviews of the service”

(Case 5)

One organisation took advantage of learning from an external expert agency for quality improvement and went further by customising their learning to fit their hospitals.

The trust has adapted the Virginia Mason system to become the XXX (Hospital name) Improvement Method. “It is transforming the way our patients move through the hospital and the way individual services redesign pathways—to take out waste and inefficiency, reduce waiting times and make the experience better for staff and patients,”

(Case 7)

5. **Emotional**: An emotional challenge to quality improvement was the initial realisation that the organisation is failing:

“Many members of staff were shocked and disappointed when the trust entered special measures. At the time, the trust was working confidently towards gaining foundation trust status. A [non-executive director]…recalls the “complete devastation” when it was announced that the trust would go into special measures.”

(Case 2)

However, this emotion was commonly reported alongside insight that there was a need for quality improvement:

“[Leads] felt “surprise and disappointment” at the news, but recognised that “there was a large element of learning and improvement to be taken from the report and its findings”

(Case 3)

Emotional responses also had the potential to impact on the quality improvement culture of the organisation:

“Staff had phenomenal stories about their improvement, but I suppose when I arrived I found quite a fear of sharing improvement”

(Case 6)

6. **Physical and technological**: A lack of physical infrastructure and IT were reported to compound flailing quality improvement endeavours:

“… the inspection period was a “perfect storm,” with issues in the new IT system, finance concerns and bed pressures, as well as a disconnect between the senior leadership team and frontline staff.”

(Case 3)

Gaining the balance between cost saving and effective governance is challenging with some organisations reporting the longer-term impact where this equilibrium had not been achieved:

“The processes and systems had been broken for some time,”…“So the financial systems and systems for setting budgets had been broken, the governance systems for managing the board, and clinical governance. All the back-office systems had been stripped out so they were at a minimal level.”

(Case 6)

However, physical infrastructure and IT can also support the resolution of other quality improvement challenges:

The ‘Happy App’ is an interactive web-based tool to gather real-time feedback from staff. They can use the app to indicate how happy they are at work and record why. The app gives managers the opportunity to monitor and understand staff satisfaction and engagement, and enables them to act on issues.

(Case 5)

7. **Structural**: The importance of putting systems in place was stressed: “We have a systematic approach to dealing with harm now and a clear reporting mechanism,” (Case 2). For example, tell Ellie:

Previously, the trust had waited for patients to come to them and in reaction they launched the Tell Ellie campaign and took staff out into the community to meet and engage with local people.

(Case 2)

Although fiscally beneficial, the lack of structure directly impacted clinical care and had to be addressed:

“The organisation should be clinically led, but clinicians need managers to help them. “We had to try to bring back a managerial structure without worsening the financial position. Addressing that problem wasn’t cost-neutral, but there was recognition from the board that the lack of structure wasn’t sustainable.”

(Case 8)

Approaches to introducing structure were reported, again demonstrating the spill-overs between the QUASER quality improvement challenges:

Breaking the Cycle Together events focused on operational problem-solving, and the trust introduced Schwartz rounds, a structured forum for staff to reflect on the emotional effects of caring for patients. The trust has used new posters and infographics in visual messages to staff, Chief Executive video briefings, safety bulletins, and the We are Proud to Care film, showcasing what […] “the compassion and commitment of all trust staff”.

(Case 5)

8. **External demands**: Responding to broader social, political, economic and contextual factors was reported at several levels. The importance of understanding the population at the planning stage was described:

“It serves three of the most populous boroughs in XXX, that have the highest percentage of people with chronic illness,” …“But we only have one major regional hospital. So it’s a question of capacity. When XXX Hospital was planned, the demographic dramatically shifted beyond the original projections. So the hospital was set up to deal with a smaller and healthier population.”

(Case 6)

Through listening to the local population, the proactive delivery of services that support the needs of community can be provided:

Focus groups provided input into addressing cultural sensitivities, and faith and ablution rooms were made available. The two local Healthwatch groups, also provided valuable information, and the Listening into Action (LiA) approach was renewed and extended across the area.

(Case 4)

However, listening is an ongoing activity:

The trust has improved its links with external organisations. “We had talks with Healthwatch, patient representative groups, councils, MPs and the press so we could provide them with reassurance about the trust,”

(Case 3)

However, the need to recognise and promote equality and diversity was also required within the workforce:

“She [chief executive] attended the British Association of Physicians of Indian Origin (BAPIO) national conference and presented the trust’s work on equality and diversity. XX thinks this was a turning point. “I think our Indian and Pakistani doctors who were there saw it as more than a token gesture.” The trust then formed an agreement with BAPIO for the development and training and established a BME network with BAPIO.”

(Case 1)

## 6. Discussion

Referring to Cusin’s [6] work on describing key elements of systems thinking, all cases refereed to improving systems, but it was not always clear how systems were being defined or what arbitrary boundaries were being used [4]. System levels for improvements seemed to range from individual services to whole hospital/sites. The eight QUASER challenges can largely be categorised as key system inputs (see Table 4), except for leadership and culture, which we also see as being influential in the transformation process; similarly, emotional challenges are likely to impact how individuals and the organisation respond to improving and transforming the system. From the analysis of the eight case studies published by the CQC, it is evident that the majority of the eight challenges from the QUASER study were present across all case studies. For example, challenges relating to leadership, culture and external demands were prominent in all cases. The role of leaders was reported as being critical in how the case organisations reacted to the initial CQC inspection and report. When viewed as an opportunity to drive change, these organisations tended to fair better. For most of the cases, a change in leadership was the catalyst to accepting the findings of the inspection reports and making improvements [13,20]. Five out of the eight case studies were explicit in the role of leaders in their transformation and improvement efforts. Effective leaders believe in improvement [13,41] and are able to clearly articulate the results they wish to see from improvement activities [42]. The importance of clinical leadership was also emphasised, with several of the case hospitals recognising the vital role of clinicians in setting the standard of what good looks like [43]. Clinical leaders can challenge and be challenged to safeguard and protect their patients and staff [43]. 

In terms of culture, for seven of the eight cases, this was a key theme within their improvement efforts. All cases referred to having a supportive approach to improvement. For some, this is about leadership being visible and supporting improvement activity within the organisation. This visibility of leaders is often termed as “going to the Gemba” and is an essential part of leading for improvement [44]. Organisational culture is being increasingly linked to quality but there still needs to be a better understanding of cultural dynamics. The relationship between culture and quality, safety and efficiency is not straightforward and the challenge for leaders is to understand which components of culture might influence which aspects of performance [45]. The engagement of staff was recognised as being critical to improvement as staff were seen by some organisations as having the answers to improving services. A shift from a culture of blame to one that celebrates success was a key theme across the case organisations. In several cases, the importance of developing a set of organisational values and behaviours that resonated with all staff was highlighted. This focus on culture indicates that the case organisations were addressing the QUASER challenge of creating an organisational culture where quality is central to clinical work and underpins all other activities [22].

The issues relating to internal politics and managing conflicts and relationships were not prominent within all cases. One case mentioned the need to gain the trust of staff and change the weight of opinion about quality improvement. All cases referred to the importance of engagement work with staff and the various mechanisms used for this such as social events, networking and fundraising along with specific initiatives such as ‘In Your Shoes’ listening events. Engagement was seen by some cases as an ongoing event and not one-off initiatives. Previous research has notably reported difficulties in engaging managers and front-line staff in improvement [46]. A recent review of clinicians’ understanding of quality and their level of engagement with quality improvement reported that there is no agreed definition or measurement, and both are influenced by their own personal beliefs, values and professional responsibilities [47].

A key characteristic to deliver high-quality care in a sustainable way is to take a systematic approach to QI capacity and capability building [48]. In terms of education and the upskilling of staff, some case organisations had implemented initiatives and/or joined external programmes to help them improve performance. For example, an initiative introduced by case 1 referred to as ‘listening into action’ involved front line staff proposing projects which would make a difference to patients. All projects needed to involve patients and staff. An example of joining an external programme was case 6 which were selected to take part in the Virginia Mason Institute programme which focuses on using Lean methods to support a patient-centred culture [49]. Others were possibly pursing their own internal programmes to increase QI capability and capacity. It is difficult from the cases to determine what scale and level of progress these programmes had reached. Previous research reported an array of methodological and educational approaches to building QI capability and capacity [50], both of which are crucial to the sustainability of QI [51,52]. It is important to note here that QI training and education are not sufficient on its own. A stronger QI culture has been found in organisations that supported a QI infrastructure (e.g., investing in staff and other resources to support QI) rather than those who just provided QI training [52]. The QUASER study [22] clearly states that there is a need to create a learning process that support continuous improvement, this suggests that ad hoc and one-off training initiatives are not enough. Having a skilled faculty to support improvement teams has also been noted as a success factor [48] in some cases in which it was unclear what learning mechanisms were in place to support their ongoing improvement activities and how far these reached within the organisations.

Structure was a prominent feature among the case organisations. This was discussed in relation to their approach to improvement and/or looking forward in terms of their plans to continue improving. Many of the cases mentioned changes in their organisational structure to support QI, including changes in leadership/executive team and board members and clinical management and governance structures particularly around clinical incidents. Having a structured, robust and agreed approach to quality improvement has been reported as a crucial component to building QI capacity and capability [48]. Such an approach should aid decision-making and problem-solving and include improvement tools such as plan–do–study–act (PDSA) cycles and root cause analysis. Most of the cases referred to having developed improvement and action plans. It was more evident in some cases that they had invested their efforts in one particular approach, such as the adoption of the Virginia Mason model in case 7.

The QUASER challenge of designing and providing physical systems and technological infrastructures that support improvement and quality of care [22] seemed less evident from the case data and possibly the most problematic for case organisations. Several cases referred to broken processes and the need to improve processes as part of their journey. Interestingly, one case used technology as a way of “crowdsourcing” staff feedback on working at the organisation. Others referred to improving communication and the availability of information, however it was not always clear how this had been achieved. Although the evidence base around context in QI is increasing, there remains limited knowledge and guidance concerning which contextual factors are most influential for whom and when [53].

All cases referred to the broader and complex factors that impacted on their improvement efforts, particularly for those cases who had been placed in special measures. This required them to focus on rebuilding and managing their reputation and rebuilding trust with some of their external stakeholders, which also included the regulator. Understanding the needs of their diverse populations was a priority for several of the cases, particularly during the early stages of their engagement work. Important external factors, over which organisations have little control, can positively and negatively influence the motivation to engage in QI [54,55].

## 7. Conclusions

The aim of this study was to provide a better understanding of what challenges organisations in healthcare face when trying to improve the quality of their healthcare systems and how they overcome them in practice. Drawing on a well-established research framework—QUASER—we examined eight case organisations described as improving their performance [20]. It is evident from our review that these cases have perhaps unknowingly attempted to address the eight challenges presented in the QUASER study. Some challenges seemed to be more prevalent in certain cases than in others. For example, all cases mentioned focusing on leadership, culture, politics, structure, emotions, and the external environment. There was less emphasis on education and physical and technological systems. The QUASER guide was designed to extend the traditional and mechanistic approaches to QI by encouraging organisations to assess their strengths and weaknesses against these challenges.

Healthcare organisations and systems looking to improve the quality of their services should consider using the QUASER guide as they pursue their quality improvement journey to address the key challenges discussed in this paper. The authors of the guide recognise that not all challenges may have been identified and may vary depending on an organisation’s QI maturity [22]. Both of these points may have impacted on this case review. However, this case analysis does provide examples of how the challenges have been addressed by those organisations that were under pressure to improve their performance. The qualitative accounts provided by senior leaders, managers and frontline workers provide real insights to how improvements were made. It seems from these accounts that many of the changes were developed at a local/hospital level, which depending on where the system boundary is drawn could be interpreted as not being at a system level (integrated care system).

To the best of our knowledge, this is the first time that the QUASER challenges were retrospectively analysed to demonstrate how these manifests within organisations that improved their performance. The study was limited by the level of data provided by the published cases. This secondary analysis of the data provides some insights as to how the organisations achieved their improvements. Although some reflections are provided in terms of the learning encountered by the case organisations, these are limited. Further research to explore the difficulties experienced and perhaps some of the failings of the way would be valuable for other organisations. Similarly, ongoing in-depth case research with those organisations rated as good and/or outstanding would provide further insight into what is required to achieve a sustainable systems approach to improving healthcare services, providing a greater understanding of where system boundaries are drawn and the context and culture in which improvements are being made.

In this paper, we only looked at one guide/framework to perform our analysis. We recognise that using other frameworks such as MUSIQ [5] in conjunction with QUASER may have introduced other factors or challenges to be considered. However, this study did provide some insight into the practical steps taken by healthcare organisations to improve their performance. We analysed these against the challenges proposed in the QUASER guide and found that all of these are present to varying degrees within these improvement journeys. The maturity of these improvement efforts is not wholly clear as the extent to which a whole systems approach was taken. These are two areas that also require further consideration.

## Figures and Tables

**Table 1:** QUASER guide to the eight challenges of quality improvement with definition [22,27].

QUASER QI Challenge	Definition
Leadership	Providing clear, strategic direction.
Political	Addressing the internal organisational politics and negotiating the conflicts and relationships surrounding any quality improvement effort.
Cultural	Giving ‘quality’ a shared, collective meaning, value and significance within the organisation.
Educational	Creating and nurturing a learning process that supports continuous improvement.
Emotional	Inspiring, energising, and mobilising people for the quality improvement effort.
Physical and technological	Designing physical systems and technological infrastructures that support improvement and quality of care.
Structural	Structuring, planning and coordinating quality efforts.
External demands	Responding to broader social, political and contextual factors.

**Table 2:** Organisation characteristics (source: [20] and data in italics is from organisations’ website).

Organisation	Population	Staff	Beds	Additional Detail
Case 1	365,000	6000	Not available	Three hospitals
Case 2	521,000	8000	1041	Two acute sites and three community sites
Case 3	578,000	10,100	1400	Two hospitals
Case 4	465,000	3400	700	Two hospitals
Case 5	500,000	13,000	Not available	Eight hospitals (largest NHS Trust in England)
Case 6	750,000	7500	Not available	Operates from two sites
Case 7	78,000 from metro area and 5.4 million from surrounding community	15,000	1785	Four inpatient sites
Case 8	380,000	4000	800	Five sites

**Table 3:** The selected case organisations’ journey from special measures (adapted from [20]). Key: SpM—special measures.

Organisation	Special Measures	Inadequate	Requires Improvement	Good	Outstanding
Case 1	June 2014	July 2015 (out of SpM December 2015)		February 2017	
Case 2	July 2013		May 2014	2015	
Case 3	September 2015			January 2017 and came out of SpM	
Case 4	February 2014			February 2016	
Case 5			December 2014		March 2017
Case 6	December 2013	March 2015	March 2017		
Case 7			July 2014	September 2016	
Case 8		April 2015		December 2016	

**Table 4:** QUASER coding identified by organisation.

Case Study	Leadership (I and T)	Political (I)	Cultural (I)	Educational (I)	Emotional (T)	Physical and Technological(I)	Structural (I)	External Demands (I)
Case 1	X	X	X		X	X	X	X
Case 2	X	X	X	X	X	X	X	X
Case 3	X	X	X		X	X	X	X
Case 4	X	X	X		X		X	X
Case 5	X	X	X	X	X	X	X	X
Case 6	X	X	X	X	X	X	X	X
Case 7	X	X	X	X	X			X
Case 8	X	X	X		X		X	X

Key: X denotes the present within the case data; I = inputs; T = transformation.

## Data Availability

The case data used in this paper is publicly available.
